# Suture-Mediated Vascular Closure Improves Same-Day Discharge in Atrial Fibrillation Ablation and Left Atrial Appendage Occlusion

**DOI:** 10.1016/j.jaccas.2026.109427

**Published:** 2026-09-16

**Authors:** Mark J. Shen, Shal T. Shen, Hannah Serrano

**Affiliations:** a Department of Cardiovascular Research, Cardiovascular Medicine, PLLC, Moline, Illinois, USA; b Department of Cardiology, UnityPoint Health-Trinity, Rock Island, Illinois, USA

**Keywords:** electrophysiology, femoral venous access, left atrial appendage occlusion, quality improvement, same-day discharge, vascular closure

## Abstract

**Background:**

Same-day discharge after atrial fibrillation ablation and left atrial appendage occlusion procedures may improve patient experience, bed availability, and procedural throughput, but prolonged bed rest remains a common operational barrier.

**Project Rationale:**

We identified the vascular closure strategy and recovery time as modifiable constraints limiting reliable same-day discharge after large-bore femoral venous access.

**Project Summary:**

A multidisciplinary team implemented a standardized suture-mediated vascular closure protocol for selected atrial fibrillation ablation and left atrial appendage occlusion procedures. In a retrospective comparison of 139 consecutive cases, suture-mediated closure was associated with shorter time to ambulation, shorter time to discharge, and a higher same-day discharge rate compared with Figure-of-8 closure, without an observed increase in vascular or bleeding complications.

**Take-Home Messages:**

Standardized suture-mediated closure may help convert same-day discharge into a predictable operational pathway. Procedural efficiency and patient safety should be measured together.

## Visual Summary

**Figure:**
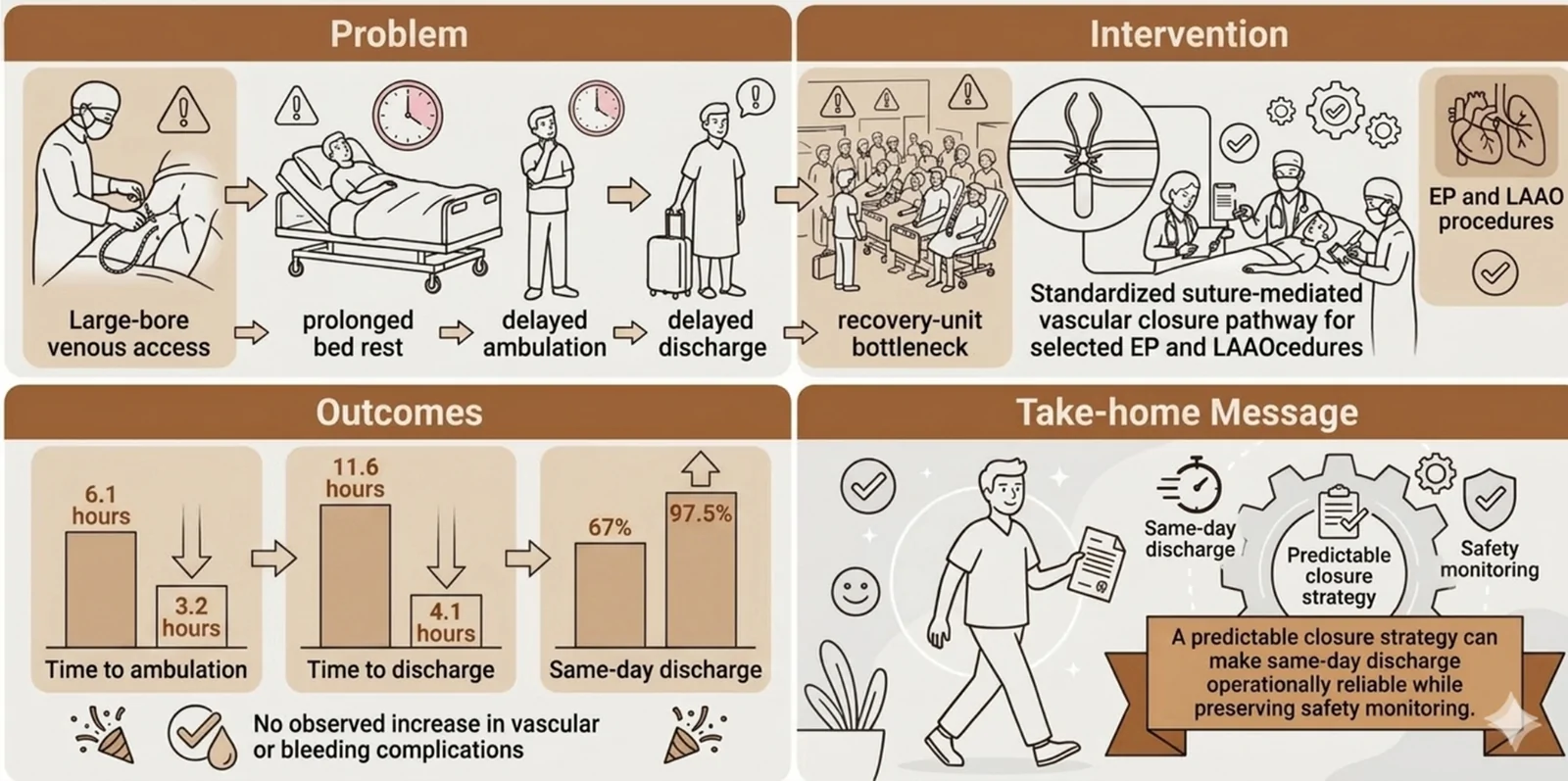

Same-day discharge (SDD) has become an increasingly important quality target in cardiovascular procedural care. Contemporary Heart Rhythm Society/American College of Cardiology guidance supports SDD for appropriately selected patients undergoing intracardiac catheter ablation when patient selection, procedural planning, postprocedural monitoring, and shared decision-making are protocolized.^1^ National registry data also show that SDD after atrial fibrillation (AF) ablation has increased substantially in the United States, while overall and major complication rates remain low among selected patients discharged the day of the procedure.^2^

**Take-Home Messages**

• A standardized suture-mediated closure pathway can substantially reduce recovery-time bottlenecks after large-bore femoral venous access procedures and support reliable same-day discharge.
• Quality improvement efforts in procedural cardiology should measure efficiency and safety together, using operational metrics such as time to ambulation, time to discharge, discharge success, and complications.

For electrophysiology (EP) procedures and left atrial appendage occlusion (LAAO), SDD is not simply a final discharge decision. It is the product of an organized workflow that begins with patient selection and extends through access-site management, nursing coordination, recovery monitoring, and discharge readiness. In many programs, prolonged postprocedural bed rest after large-bore femoral venous access remains a practical bottleneck.

**Visual Summary:**
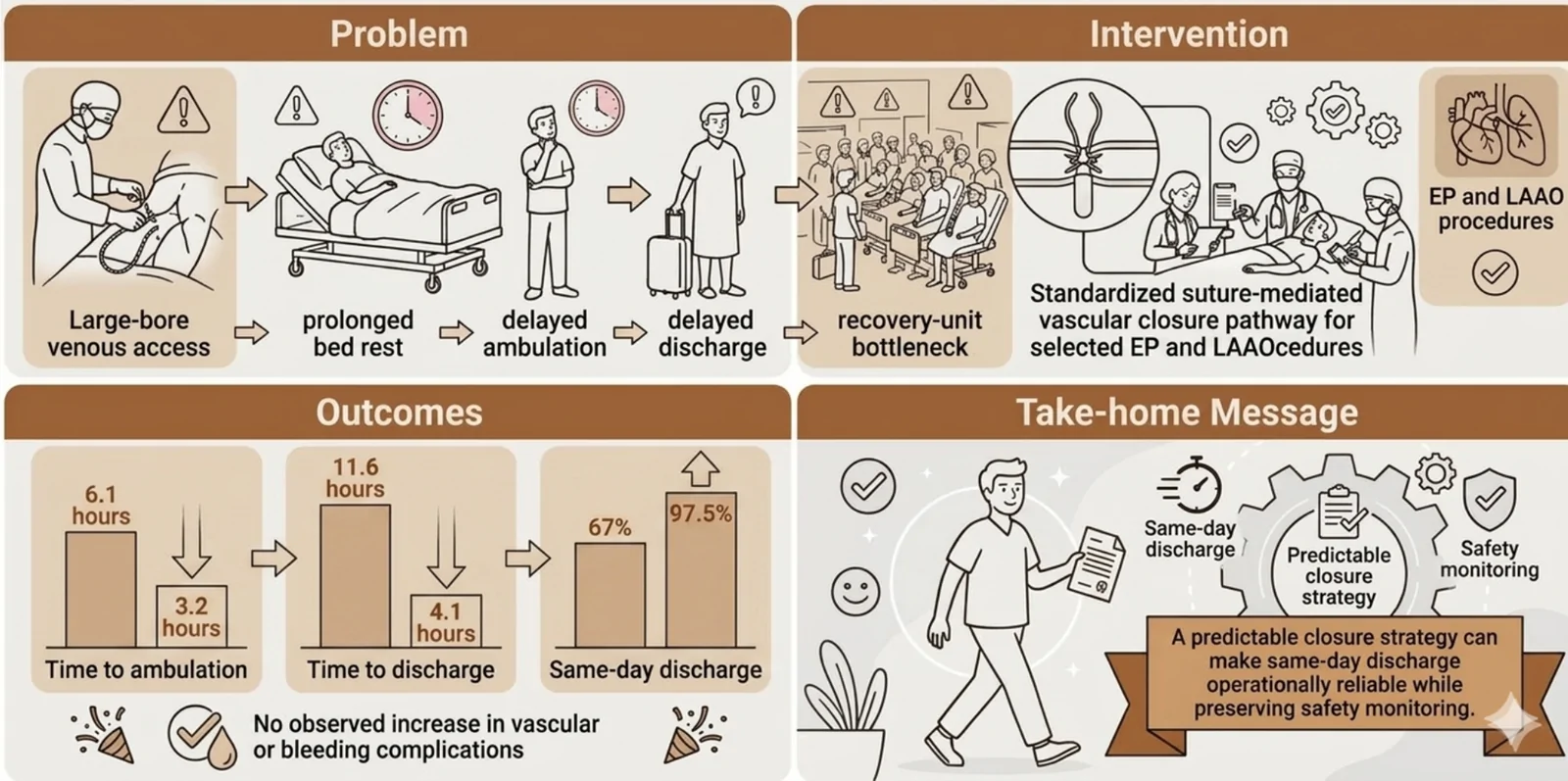
Removing the Bedrest Bottleneck in EP and LAAO Recovery The visual summary illustrates the quality improvement pathway from problem identification to intervention and outcomes: large-bore venous access led to prolonged bed rest, delayed ambulation, and delayed discharge; a standardized suture-mediated vascular closure pathway was implemented for selected electrophysiology (EP) and left atrial appendage occlusion (LAAO) procedures; and workflow outcomes improved, including shorter time to ambulation, shorter time to discharge, and increased same-day discharge, without an observed increase in vascular or bleeding complications.

This project addressed that operational constraint by standardizing access-site closure. We evaluated whether a suture-mediated closure (SMC) pathway could shorten time to ambulation, shorten time to discharge, and improve SDD feasibility compared with a traditional Figure-of-8 closure approach.

## Case Summary Prompting the Project Launch

Our EP and LAAO program faced increasing procedural volume and pressure on recovery resources. Although many patients were clinically appropriate for SDD, prolonged bed rest after femoral venous access frequently delayed ambulation and discharge readiness.

Internal workflow review suggested that the closure strategy was a major contributor to this bottleneck. Figure-of-8 closure and manual compression workflows often required extended observation before ambulation. This delay affected individual recovery, day-surgery throughput, bed availability, staff coordination, and predictability of discharge planning. The project was launched as a quality improvement initiative to determine whether a standardized SMC pathway could safely accelerate recovery after large-bore venous access procedures.

## Project Rationale

The rationale was based on a simple operational premise: if vascular hemostasis can be made more reliable and ambulation can occur earlier, then SDD becomes more feasible and predictable. This premise aligns with prospective SDD studies showing that standardized eligibility criteria and coordinated postprocedural pathways can support safe discharge after AF ablation.^3^^,^^4^

Large-bore venous access closure is also an important area for workflow innovation. The suture closure AFtEr large bore vein access (SAFE-VEIN) study found that Perclose ProGlide (Abbott Laboratories) reduced time to hemostasis and time to ambulation compared with the Figure-of-8 suture after large-bore venous access.^5^ Another randomized trial in LAAO and transcatheter mitral repair patients found both Perclose ProGlide and Figure-of-8 closure feasible and safe, underscoring that the closure strategy should be evaluated not only for complications but also for operational endpoints and cost.^6^ These considerations are especially relevant as contemporary LAAO practice expands and large registry data demonstrate high implant success and favorable in-hospital safety with WATCHMAN FLX (Boston Scientific Corporation) in routine practice.^7^

## Project Description

A multidisciplinary workgroup was assembled that included electrophysiologists involved in LAAO, catheterization laboratory and EP nurses, postanesthesia care unit staff, and cardiac treatment unit recovery personnel. The team developed a standardized closure pathway for selected EP and LAAO procedures requiring large-bore femoral venous access, generally 12- to 16-F. SMC was designated as the preferred strategy for eligible patients, with the goal of achieving rapid hemostasis and reducing mandatory bedrest duration.

This retrospective study reflects a continuous roll-in experience in our EP laboratory during the implementation of SMC. Before SMC was introduced, consecutive eligible patients were managed using the existing Figure-of-8 closure approach. After Perclose ProGlide became available in the EP laboratory, subsequent consecutive eligible patients were managed according to the SMC pathway. Thus, the closure strategy was determined by the timing of the practice change rather than by case-by-case operator preference. The comparison therefore represents 2 sequential workflow periods: the preimplementation Figure-of-8 period and the postimplementation SMC period.

We then performed a retrospective comparative analysis of 139 consecutive cases involving pulsed field ablation and LAAO. The cohort included 81 cases managed with SMC and 58 cases managed with Figure-of-8 closure. Primary workflow outcomes were time to ambulation, time to discharge, and SDD rate. Safety outcomes included vascular and bleeding complications. Electronic health record timestamps were used to measure recovery intervals and discharge timing. Continuous variables were summarized as mean ± SD, and categorical variables were summarized as percentages. Comparisons between the SMC and Figure-of-8 groups used Welch 2-sample *t*-tests for continuous variables and Fisher exact tests for categorical variables. Non-normally distributed workflow outcomes were additionally assessed using Mann-Whitney *U* testing. Safety outcomes were summarized descriptively because complication rates were low. All tests were 2-sided, with statistical significance defined as *P* < 0.05.

## Project Deliverables

The project produced 4 practical deliverables. First, it established a standardized large-bore venous closure pathway for selected EP and LAAO cases. Second, it created shared workflow expectations among procedural physicians, nurses, and recovery staff. Third, it generated measurable quality improvement indicators: time to ambulation, time to discharge, SDD success, and access-site safety. Fourth, it supported a reproducible outpatient-first framework that can be refined prospectively and expanded to additional procedure types, operators, or sites.

## Project Outcome, Impact, and Future Directions/Next Steps

Among 139 consecutive procedures, SMC was associated with significant improvements in all primary workflow outcomes. Compared with Figure-of-8 closure, SMC was associated with a shortened time to ambulation from 6.1 ± 1.3 hours to 3.2 ± 1.1 hours, a 47.5% reduction (*P* < 0.001). Time to discharge decreased from 11.6 ± 7.1 hours to 4.1 ± 2.8 hours, a 64.6% reduction (*P* < 0.001). SDD increased from 67.0% to 97.5% (*P* < 0.001).

These operational gains were not associated with an observed increase in vascular or bleeding complications. Earlier ambulation created a more predictable discharge window, improved recovery-unit flow, and supported a more reliable outpatient-first model for appropriate patients. Because this analysis was retrospective and reflects early postimplementation experience, these findings should be interpreted as hypothesis-generating and as the basis for prospective protocol development rather than as definitive evidence of system-wide benefit. Future work will prospectively evaluate not only procedural safety and recovery intervals, but also total length of stay, SDD feasibility, patient-reported experience and satisfaction, staff workflow burden, resource utilization, and direct and indirect cost savings. These future analyses will inform refinement of standardized eligibility criteria, recovery milestones, discharge criteria, postdischarge follow-up, and broader multicenter assessment across different staffing models and recovery-unit structures.

## Discussion

This project demonstrates how a focused procedural workflow change can produce measurable gains in care delivery. The central problem was not a lack of clinical interest in SDD, but a lack of a predictable operational pathway to achieve it. By targeting access-site management, the team addressed one of the most important modifiable constraints in recovery.

The improvement in time to ambulation was particularly meaningful because ambulation is often the practical gateway to discharge readiness. When bedrest duration is prolonged, discharge becomes vulnerable to downstream delays, including staffing availability, recovery-unit congestion, transportation timing, and patient fatigue. By shortening the bedrest interval, SMC made the postprocedural course more predictable.

The project also illustrates the importance of interdisciplinary implementation. The intervention required physician procedural adoption, nursing confidence in access-site assessment, recovery-unit coordination, and agreement on discharge expectations. The device was therefore only one component of the improvement; the larger intervention was the standardized pathway surrounding device use.

Several limitations should be acknowledged. This was a retrospective, single-operator, single-center comparison during the initial implementation of SMC in our EP laboratory. Other operators were not included in the initial analysis because they maintained their existing closure practices during the early implementation period. As a result, these findings may reflect operator-specific workflow, technique, patient-selection factors, procedure type, and temporal changes in workflow and may not be fully generalizable to all operators or practice settings. The study was not randomized, and rare complications may not be fully captured in a modest sample. Patient-reported outcomes, staff workload measures, and formal cost analyses were not included in the initial evaluation. In addition, the project should be interpreted within the broader AF and LAAO evidence base, in which guideline-directed patient selection and anticoagulation decision-making remain central to procedural planning.^8^

## Conclusions

A standardized SMC protocol for large-bore femoral venous access was associated with shorter time to ambulation, shorter time to discharge, and a higher SDD rate after EP and LAA closure procedures. These gains were achieved without an observed increase in vascular or bleeding complications.

For programs seeking to expand SDD, access-site management should be viewed as a key operational lever. SMC may help transform SDD from an inconsistent outcome into a reliable, protocol-driven pathway.

## Funding Support and Author Disclosures

The authors have reported that they have no relationships relevant to the contents of this paper to disclose.
